# Tracking the evolution of virtual reality applications to rehabilitation as a field of study

**DOI:** 10.1186/s12984-019-0552-6

**Published:** 2019-06-21

**Authors:** Emily A. Keshner, Patrice Tamar Weiss, Dorit Geifman, Daphne Raban

**Affiliations:** 1 Department of Physical Therapy, College of Public Health, Temple University, Ritter Annex Room 683, 1301 Cecil B. Moore Ave, Philadelphia, PA 19122 USA; 2 Department of Occupational Therapy, Faculty of Social Welfare and Health Sciences, University of Haifa, Haifa, Israel; 3 Faculty of Management, University of Haifa, Haifa, Israel

**Keywords:** Virtual rehabilitation, Topic modeling, Interdisciplinary, Scientific communities

## Abstract

**Background:**

Application of virtual reality (VR) to rehabilitation is relatively recent with clinical implementation very rapidly following technological advancement and scientific discovery. Implementation is often so rapid that demonstrating intervention efficacy and establishing research priorities is more reactive than proactive. This study used analytical tools from information science to examine whether application of VR to rehabilitation has evolved as a distinct field of research or is primarily a methodology in core disciplines such as biomedical engineering, medicine and psychology.

**Methods:**

The analysis was performed in three-stages: 1) a bibliographic search in the ISI Web of Science database created an initial corpus of publications, 2) the corpus was refined through topic modeling, and 3) themes dominating the corpus from the refined search results were identified by topic modeling and network analytics. This was applied separately to each of three time periods: 1996 to 2005 (418 publications), 2006 to 2014 (1454 publications), and 2015 to mid-2018 (1269 publications).

**Results:**

Publication rates have continuously increased across time periods with principal topics shifting from an emphasis on computer science and psychology to rehabilitation and public health. No terminology specific to the field of VR-based rehabilitation emerged; rather a range of central concepts including “virtual reality”, “virtual gaming”, “virtual environments”, “simulated environments” continue to be used. Communities engaged in research or clinical application of VR form assemblages distinguished by a focus on physical or psychological rehabilitation; these appear to be weakly linked through tele-rehabilitation.

**Conclusions:**

Varying terms exemplify the main corpus of VR-based rehabilitation and terms are not consistent across the many scientific domains. Numerous distinguishable areas of research and clinical foci (e.g., Tele-rehabilitation, Gait & Balance, Cognitive Rehabilitation, Gaming) define the agenda. We conclude that VR-based rehabilitation consists of a network of scientific communities with a shared interest in the methodology rather than a directed and focused research field. An interlinked team approach is important to maintain scientific rigor and technological validity within this diverse group. Future studies should examine how these interdisciplinary communities individually define themselves with the goals of gathering knowledge and working collectively toward disseminating information essential to associated research communities.

## Background

Virtual Reality (VR) in general, and the application of VR to rehabilitation in particular, is a relatively young, interdisciplinary field where clinical implementation very rapidly follows scientific discovery and technological advancement. Indeed, implementation is often so rapid that demonstration of intervention efficacy by investigators, and establishment of research and development priorities by funding bodies, tends to be more reactive than proactive.

Rapid growth in the number and type of applications of VR to rehabilitation has occurred over the past 15 years, suggesting that the research in this area may be demonstrative of a new scientific field. Reviews of the research in this area (see e.g., Rizzo and Kim [1], Sveistrup [2], and Levin et al. [3]), however, focus principally on applications of VR technology to specific disability or impairment. If VR-based rehabilitation is chiefly one more tool in the field of rehabilitation science, then cross-disciplinary communication could consist primarily of reporting methodological approaches. If, however, VR-based rehabilitation has emerged as a distinctive scientific domain, then it becomes the responsibility of the scientists and clinicians engaged in this work to disseminate both research insights and future directions across engaged disciplines. Our aim in the current study is to use tools of analysis from the domain of information science to examine whether application of VR to rehabilitation has evolved as a distinct field of research or is primarily a methodology in core disciplines such as biomedical engineering, medicine and psychology.

**Fig. 1:**
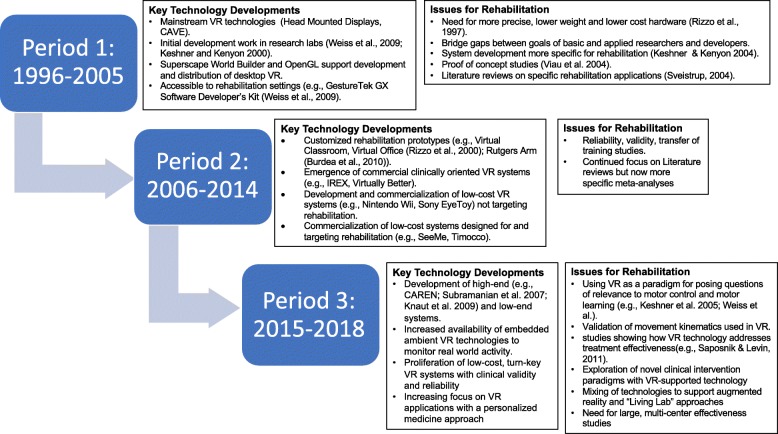
Key technological developments that influenced the use of VR technology in rehabilitation

We initiated our search in 1996 because only one moderately relevant review article alluding to virtual reality being applied to medicine was found prior to that time [4]. Thus Period 1 (1996–2005) is defined as the period in which key technological developments emerged that influenced the use of VR technology for rehabilitation (Fig. 1). The most characteristic features of the early technologies in Period 1 were their large size, high cost and limited accuracy. These systems led to several pioneering motor rehabilitation applications [5–9] whose clinical relevance was still uncertain since their high cost, technical complexity, and encumbrance severely limited access to both hardware or software [10]. Although there was limited recognition of its growing clinical potential, no significant grassroots perception of the need for VR-based interventions took hold during this period.

Key changes over the period between 1996 and 2005 included the emergence of platforms such as Superscape World Builder and OpenGL that supported easier development and distribution of desktop VR applications [11]. During this period, the first clinically oriented commercial VR systems emerged such as IREX (for motor rehabilitation) and Virtually Better (for treatment of phobias) appeared. The focus of the work was clearly research-oriented since only funded groups could support the creation of customized rehabilitation prototypes (e.g., virtual classroom [12] and haptic interfaces [13]). VR technology began to be directed to specific rehabilitation applications, following the Rogers and Everett’s [14] pattern of Diffusion of Innovations where the innovators directly interacted with and motivated early adopters of the new technology. An example of this collaboration is that of researchers at the Rehabilitation Institute of Chicago and at the Electronic Visualization Laboratory at the University of Illinois at Chicago (https://www.evl.uic.edu/) to perform kinematic analyses of body kinematics in the CAVE with an eye toward identifying the impact of visual perception on postural control [15–17]. Another example is parallel work at the Hebrew University, University of Haifa and University of Ottawa to recommend refinements and experimental data that would make the GX/IREX VR systems more accessible for rehabilitation purposes [18].

Period 2 (2006–2014) was shaped by development and initial implementation of clinically accessible applications. For example, this period witnessed the development and commercialization of both high-end (e.g., CAREN, Motekforce, Netherlands) and low-cost VR systems as the early adopters began to recognize the value of VR to clinical treatment [19]. Off-the-shelf (e.g., Nintendo Wii, Sony EyeToy) products that did not target rehabilitation began to be widely used by clinicians because of their accessibility and low cost. Some clinical centers designed an own software or used external props as a compensation for commercial games that were not appropriate for persons with neuromuscular impairments. More recently, and in particular since 2010, a number of low-cost VR systems designed for and targeting rehabilitation (e.g., SeeMe, Timocco, Kinect, VAST, VAP-S) have become available. A variety of rehabilitation-oriented desktop gaming programs that implement VR properties (e.g., feedback, documentation, motivation) also became increasingly available.

Finally, Period 3 (2015–2018) was defined by the present stage of refinement of meaningful clinical research. For example, since 2015, the increasing accessibility of embedded ambient technologies (e.g., inexpensive cameras, proximity sensors, wearable computing) that support the monitoring of motor and cognitive functioning under real-world conditions has extended VR-based interventions beyond the clinical setting. One of the most exciting recent trends is the availability of authoring tools that enable clinicians to program virtual environments with relative ease (e.g., [20]). Another recent development is the use of personalization that enables automatic adjustments of levels of difficulty as a client performs tasks in virtual environments (e.g., [21]). Lastly, there has been an upsurge in the availability of high fidelity yet reasonably costing head-mounted display devices that promote the use of immersive VR in a range of clinical settings [22].

From our approach to classifying these three periods, it is tempting to conclude that progress in the rehabilitation focus of this field was completely reliant on technological advances (Fig. 1). This point of view, however, ignores the queries of the scientific disciplines that have shaped the direction of these technological advances. Similar to other rehabilitation technologies, the field of VR-based rehabilitation has emerged as a result of true interdisciplinary and interprofessional collaborations between basic science, clinical science, and industry [23]. Multiple fields of study have played vital roles in extending our knowledge base, and in determining which technological developments have been critical for assisting the profusion of present day applications. A recent paper by Cipresso et al. [24] applied tools of network analytics to study the fields of virtual reality and augmented reality from 1970. Although they did not include the term “rehabilitation” in their search, they found that the field was expanding and moving toward increasing clinical applications.

We are the first to analyze the domain specific terminology using a topic modeling method to explore the foundation of this broad, multidisciplinary field. Topic modeling, specifically Latent Dirichle Allocation (LDA), is a text mining method that identifies latent topics and themes in a large corpus of text that consists of many documents [25]. It is used to navigate through large archives [26], enhance Information Retrieval (IR) methods [27], identify latent topics within repositories of documents by using a synchronic approach, and study the dynamics of knowledge development over time [28]. Unlike systematic or scoping reviews of the quality of publications in a field, topic modeling provides a quantitative exploratory text analysis of the academic literature [29]. We now examine the structure and citation patterns of VR-based research by complementing the topic modeling method with network analysis [30].

In a previous paper, we investigated the interdisciplinary nature of the field of VR-based rehabilitation by studying the patterns of academic publications in the VR-based rehabilitation literature over the past 22 years [31]. We concluded that the field is evolving from a predominantly technology development effort to a focus on how technology can support rehabilitation principles and outcomes. In the present paper we have analyzed a corpus of academic publications that include both technology development and the clinical applications guiding this field. The questions we have attempted to address through this analysis are: 1) Is there a consistent terminology reflecting specific content areas (i.e., topics) in the field of VR-based rehabilitation? 2) Do these terms emerge across the corpus of research performed with VR? 3) Does VR-based rehabilitation emerge as a multidisciplinary field or as multiple disciplines utilizing a common technology? 4) Are there distinguishable research and clinical foci that would define future directions for this field? Our larger goal is to identify the themes appearing within VR-based rehabilitation in order to determine whether the full potential of this technology is being recognized and accessed [32–34].

## Methods

LDA [25] uses a generative, unsupervised machine-learning algorithm that refines the semantic classification of a document beyond the rudimentary analysis of its raw terms. This allows for a more subtle topic analysis as raw terms may assume a different meaning subject to the context of their usage. The method builds on the assumption that documents maintain a latent structure of topics and that each topic is a distribution over a fixed vocabulary of the whole corpus; therefore, each term is analyzed in the context of the document in which it appears. The method is an exploratory method and it does not require a priori annotation or labeling of the documents or terms within the documents. Instead, the algorithm infers the hidden topic structure of the corpus by computing the conditional distribution of the hidden variables (the topic structure) and the observed variables (the words of the documents).

**Fig. 2:**
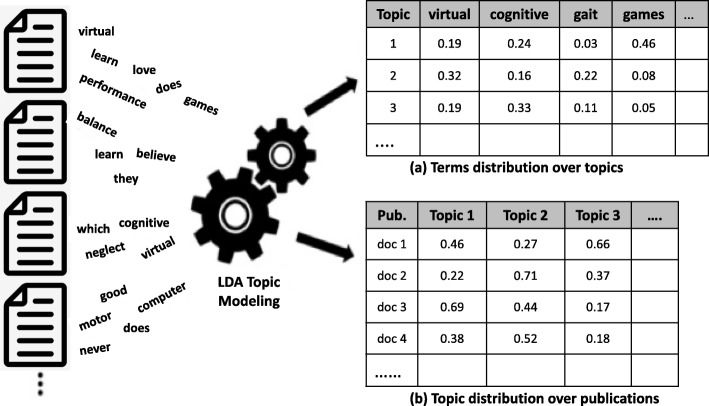
A schematic demonstration of the LDA topic modeling process schematically demonstrates how the LDA algorithm processes words that are included in a large corpus of publications in the field of VR-based rehabilitation (left of diagram). **a** Terms distribution over topics. **b** Topic distribution over publications

The thematic structure of the document is identified by calculating the proportion of representation of each topic within the document. Figure 2, based on Raban & Geifman [30], schematically demonstrates how the LDA algorithm processes words that are included in a large corpus of publications in the field of VR-based rehabilitation to produce a dual outcome: (1) a table of topics, each identified by the probability distribution of all the raw terms in the corpus (Fig. 2a), and (2) for each publication, the estimated distribution of the topics running through them, i.e. the degree of which each topic is represented in the document (Fig. 2b).

Topic modeling is an exploratory method that requires much iteration in order to reach meaningful results. This study employed a three-stage analysis to obtain results from the topic modeling: Stage 1) a bibliographic search in the ISI Web of Science (WoS) database to create an initial corpus of documents; Stage 2) refining the bibliographic search results through topic modeling; and Stage 3) identifying the themes that dominated the research agenda of the field by applying topic modeling on the refined search results.

There are various quantitative approaches to reach the desired outcome, however, quantitative methods may generate less semantically meaningful topics [35]. Thus we chose to use a computational approach vetted by the domain experts wherein each iteration cycle was reviewed, and an outcome that was considered to best reflect the predominant research areas in the field was selected.

### Stage 1: bibliographic search

The initial search was performed in WoS. Search terms included “rehabilitation” both as a topic and as a WoS category, combined with “virtual reality” or “virtual environment” or “simulation” and were then refined by excluding areas considered to be irrelevant to the field of interest: water resources, environmental sciences, ecology, automation control systems, construction building technology, materials science, mathematical computational biology, agriculture, energy fuels, mechanics, operations research management science, mathematics, physical geography, physics, cell biology, business economics, fisheries, forestry, geography, thermodynamics, metallurgy metallurgical engineering, mining mineral processing, nuclear science technology, criminology penology, meteorology atmospheric sciences, archaeology, film radio television, geochemistry geophysics, oceanography.

Results of a series of iterative searches were evaluated by the two domain experts who concluded that a strictly automated search did not uncover many of the important publications and established investigators in the field. We, therefore, chose to perform a broader search and to use the topic modeling approach to aid in further filtering of the results. The rationale was to make the search as broad as possible, yet avoid the inclusion of non-relevant areas of research cited in the exclusion list above. The resultant bibliographic search generated a corpus of 3131 papers across the three periods.

### Stage 2: refining the search results

The purpose of Stage 2 was to refine and filter the data set. Topic modeling was used to remove topics and, hence, papers that were marginally relevant to the core domain of VR-related rehabilitation. Due to a continuously increasing number of publications over time, we found that analyzing all years of interest as a single period created a bias towards research areas that dominated the later years of the search. As shown in Table 1, and as described in the introduction section, we divided the search range into three periods, Period 1 (P1), from 1996 to 2005 (418 publications), Period 2 (P2), from 2006 to 2014 (1454 publications) and Period 3 (P3), from 2015 to mid-2018 (1269 publications). The algorithm was applied independently to each period. For each period we created a corpus of text that consisted of the publication title, abstract, and all keywords available in WoS. The topic modeling algorithm determined whether a publication was representative of a specific topic (i.e., if the topic is ranked highest on its distribution list as seen in Fig. 2b). The method provides a probability distribution of the topics that are discussed within each publication and we used the topic with the highest probability as the publication’s topic. Other topics may also be represented at a lower (and sometimes close) probability.

**Table 1:** Topics in the three periods identified by using the topic modeling algorithm. Topics in bold are those removed from the analysis. “N” is the number of publications per topic

Topic	Period 1: 1996 to 2005 (Total *N* = 418)		Period 2: 2006 to 2014 (Total *N* = 1454)		Period 3: 2015 to mid-2018 (Total *N* = 1269)	
	Topic Name	N	Topic Name	N	Topic Name	N
1	**Muscle Stimulation & Gait**	**29**	**Robotics & External Devices**	**130**	**Orthopedics & Biomechanics**	**125**
2	**Civil Engineering**	**23**	Gait	100	Gait and Balance	68
3	**Surgery & Medicine**	**14**	Motor Imagery	92	**Orthopedics & Civil Engineering**	**68**
4	Technology for Rehabilitation	27	Technology for Rehabilitation	105	Robotics for Upper Extremity Rehabilitation	115
5	**Biomechanics**	**23**	**EMG, Neural Transmission and Simulation**	**86**	**EMG, Neural Transmission and Simulation**	**71**
6	Rehabilitation Interventions	76	Vision and Driving Simulations	88	**International Health Care**	**67**
7	**Wheelchair and Driving Simulations**	**20**	**Speech, Language and Hearing**	**117**	Gaming	76
8	Assistive Technologies	37	General Gaming	76	Measurement for Assessment	56
9	Computer Based Simulation	23	**Civil Engineering**	**85**	Gaming	78
10	Simulation for Functional Assessment and Teaching	31	Upper Extremity Rehabilitation	120	**Simulation for Health Care Education**	**90**
11	Ergonomics and Simulation	21	Cognitive Rehabilitation Assessment and Intervention	89	Assessment and Intervention	74
12	**EMG, Neural Transmission & Simulation**	**30**	**Orthopedic Biomechanics**	**72**	Meta-Analyses	82
13	VR-based Rehabilitation	15	Gaming	114	Cognitive Rehabilitation	93
14	**Orthopedics and Gait**	**25**	**Computerized Adaptive Testing**	**61**	Motor Rehabilitation	119
15	**Speech, Language & Hearing**	**24**	**Muscle Biomechanics**	**119**	**Motor Imagery**	**87**

The 20 most representative publications as well as the 20 highest ranking terms per topic (Fig. 2a) were reviewed by the domain experts to annotate each topic with a thematic name, and to identify the topics most relevant to that field of research (Table 1). After running several cycles of the algorithm, each with different parameters, and reviewing the outcomes, we found that distinguishing 15 topics per period was the best representation of the topical structure of the corpus.

Topics shown in bold in Table 1 were considered by the domain experts to be of low relevance to the field of VR-based rehabilitation. Of the 21 remaining topics, 1775 representative publications were extracted for the final stage of analysis. After evaluating this outcome, an additional 39 publications that were highly cited in the field and for which the probability of their second ranking topics was close in value to the probability of their first ranking topics were included. The resultant 1814 relevant papers consisted of 231 publications in Period 1 (1996–2005), 807 publications in Period 2 (2006–2014) and 776 publications in Period 3 (2015 – mid-2018).

### Stage 3: refining topics and trends that dominated the research agenda

The purpose of Stage 3 was to study the patterns of emergence of the field from the most germane publications. In order to generate a set of topics common across all periods, the topic modeling algorithm was applied to the corpus as a whole without partitioning it into the three separate periods. We were then able to identify temporal trends within the topics over the three periods. Following several iterations, the domain experts determined that 10 topics represented an optimal partitioning of the corpus into its thematic structure (Table 2). As a result, by using the process described in Stage 2, each of the publications in the entire corpus could be assigned to one of the topics based on its highest ranked probabilities. Domain experts then assigned a thematic name to each topic based on the 20 terms and 20 publications ranked highest for each of the 10 topics.

**Table 2:** Topic themes based on terms, titles, keywords and absracts of the 20 most relevant papers. N is the number of publications per topic

Topic	# Publications
Cognitive issues	230
Tele-Rehabilitation	226
Simulation	140
Psychological issues	105
Neural impact	226
Gait & Balance	190
Perception/ Navigation	162
Gaming	169
Neurological conditions	133
Interventions	233

**Fig. 3:**
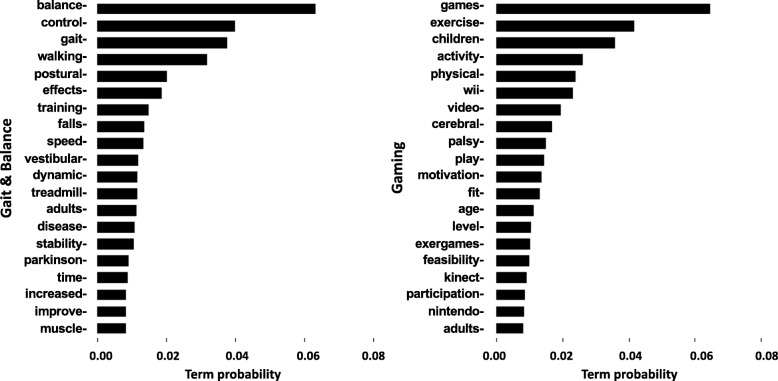
Probability distribution of 20 highest ranking terms in each of two topics

The probability distributions of the 20 highest ranking terms in two sample topics, “Gait & Balance” and “Gaming” (Fig. 3), demonstrate how the probabilistic allocation of terms to topics (cf. Fig. 2a) can assist in determining topic themes. For example, domain experts assigned the theme name “Gait & Balance” to the topic on the left as it includes the terms balance, control, gait and walking as the highest ranking terms. Gaming was assigned as the theme name to the topic on the right. Although gaming is used in many scientific disciplines (e.g., computer science, medicine), we relate to this term as having therapeutic and entertainment features (i.e., games, exercise, and activity) as the highest ranking terms. Probability distributions for all ten topics are presented in a tabular form in Appendix.

To depict the patterns of interaction among the different disciplines participating in the field of VR-based rehabilitation research, we built a network of cross-citations among all publications in the corpus. Each node in the network represents a publication from the corpus and its color represents the topic that it is best defined by. An edge links node “A” to node “B” if publication “A” cites publication “B”. The size of the node reflects the number of citations for that publication within our corpus (also known as node in-degree). To ease the visualization of the graph, only publications (nodes) that have been cited over 8 times have been included. We note that the selection of an 8 citation threshold for the displayed data did not change the patterns portrayed in the full network.

## Results

**Fig. 4:**
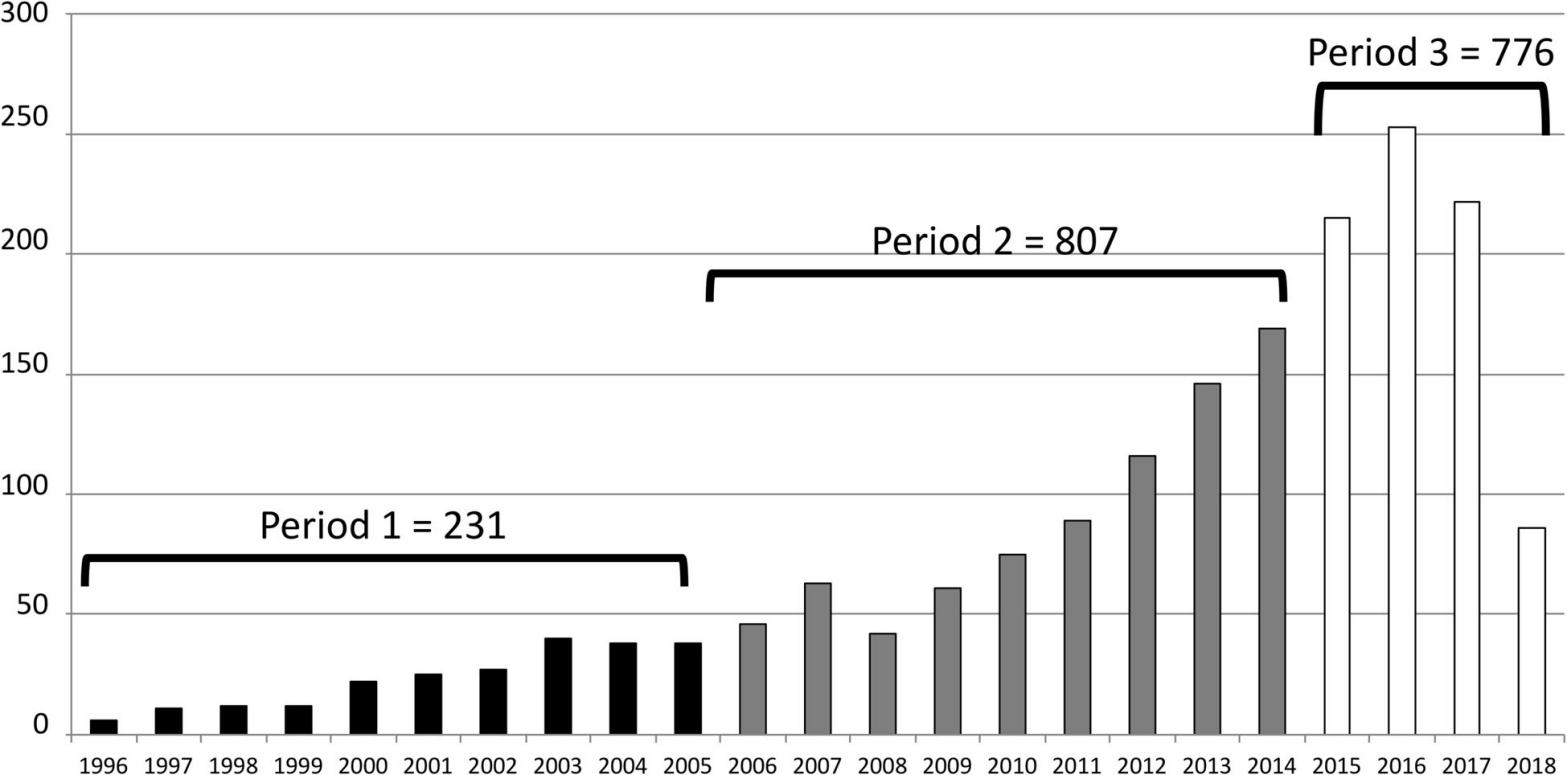
Number of publications per year across the 3 periods. Note that Period 3 includes only the first 6 months of 2018

The final corpus of 1814 publications represents the outcome of the topic modeling analysis. This refined list of publications across the three periods of interest (Fig. 4) indicates a progressive increase in the number of publications over time; even though its duration was less than half as long, Period 3 produced almost as many papers as Period 2.

### Identifying content areas in VR-based rehabilitation

We started by examining the relevant content of the field through an analysis of the Subject Categories (SC) assigned by the WoS database for these publications. In the WoS database, 252 SCs describe all of the scientific areas covered and that are applied in the process of cataloging the journal titles indexed by the database. A total of 66 SCs emerged from the bibliographic analysis of our retrieval set, however, some of these SCs were not related to psychological, cognitive or physical rehabilitation but to clinical medicine (e.g., “dermatology”) and technology development (e.g., “telecommunications”).

To illustrate the utility of the SCs, the two domain experts selected the 10 most relevant SCs in the field of VR-based rehabilitation which included 80% (2478 out of 3131) of all the SCs assigned to the publications in the corpus (Table 3). The SC “Rehabilitation” was more pronounced than other SCs for all periods accounting for more than one third of publications. The SC “Neurosciences and Neurology” accounted for only 10% of publications during Period 1 but increased to 20–25% during Periods 2 and 3. “Engineering” accounted for 8.4% of publications during Period 1 and then increased to about 12% during Periods 2 and 3. In contrast, “Computer science” declined from about 12% during Period 1 to less than 6% during Periods 2 and 3.

**Table 3:** Number of and percentage of publications in the 10 most relevant WoS Subject Categories for the total study period and for each of P1, P2 and P3

WoS Subject Categories	Total		P1		P2		P3	
	N	%	N	%	N	%	N	%
Rehabilitation	919	37.1	126	39.3	432	36.8	361	36.7
Neurosciences & Neurology	532	21.5	33	10.3	294	25.1	205	20.8
Engineering	283	11.4	27	8.4	144	12.3	112	11.4
Psychology	173	7.0	38	11.8	78	6.6	57	5.8
Sport Sciences	145	5.9	28	8.7	69	5.9	48	4.9
Computer Science	137	5.5	39	12.1	42	3.6	56	5.7
Health Care Sciences & Services	105	4.2	9	2.8	37	3.2	59	6.0
Orthopedics	67	2.7	5	1.6	32	2.7	30	3.0
Education & Educational Research	60	2.4	16	5.0	17	1.4	27	2.7
Public, Environmental & Occupational Health	57	2.3	0	0.0	28	2.4	29	2.9
Total	2478	100.0	321	100.0	1173	100.0	984	100.0

**Fig. 5:**
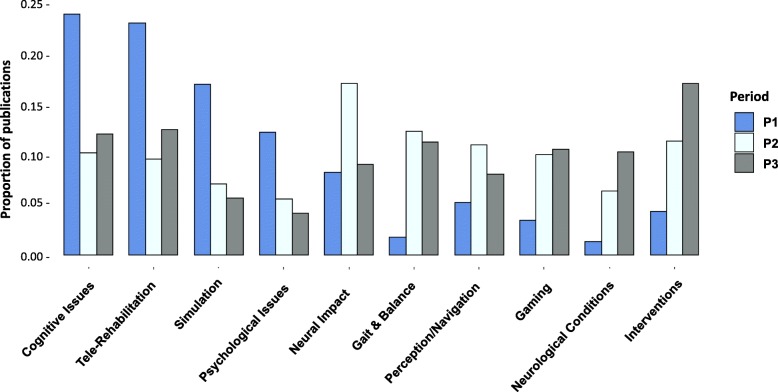
Proportion of publications for each topic in each period

Whereas SCs are typically assigned by librarians, in this study we have used text analytics to identify the emergent key topics that are researcher-driven and to examine the distribution of publications per topic across the three periods of interest (Fig. 5). We note that the topics of Simulation, Tele-rehabilitation and Cognitive Issues are prominent in Period 1, but their proportion declines greatly in subsequent periods. There is a dramatic shift in interest in Period 2 to the topic of Neural Impact whereas Period 3 demonstrates an increased interest in Intervention. In general, publications, and hence research, appear to be more evenly distributed across the ten topic themes in Periods 2 and 3 than it was in Period 1. As can be seen, topic modeling displays a more precise definition of the themes used in the research, and provides a subtler view of the trends the dominate the research across the three periods.

### Correspondence of terms across the corpus

**Fig. 6:**
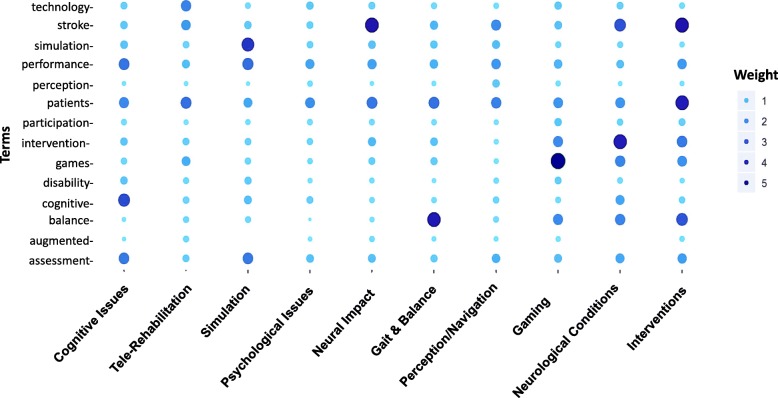
Weighted frequency of important terms over the 10 topics

To gain insight into terminology representing each of the topics, the two domain experts selected terms that were considered to be particularly meaningful to the field of VR-based rehabilitation. The weight (the ratio of the frequency of terms in a given topic to the number of representative publications) of these terms was compared across each of the topics (Fig. 6). For example, the topic of Gaming is clearly identified by the term “games” which also appears frequently in the Intervention, Neurological Conditions, and Telerehabilitation topics. The term “balance” is also weighted strongly in these same topics as well as in the topic Gait & Balance. The term “assessment” is weighted similarly for the Interventions, Simulation, and Cognitive Rehabilitation topics whereas the term “intervention” emerged in the Interventions, Gaming, and Neurological Conditions topics. The term “technology” is most frequently employed in the Telerehabilitation, topic and the terms “disability” and “participation” are not particularly weighted in any topic. The terms “patients” and “performance” appear across most topics with similar weighting.

### Multidisciplinary field or shared technology?

**Fig. 7:**
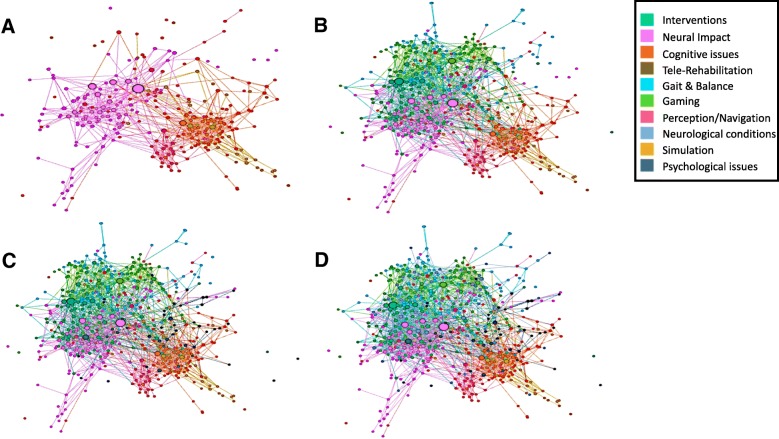
The citation network. **a** The network of publications from the topics of Cognitive Issues, Perception and Navigation, Simulation, and Neural Impact demonstrating the two assemblages. **b** Topics of Gait and Balance, Interventions, and Gaming added to the network portrayed in (**a**). **c** The addition of Psychological issues to (**b**). **d** The complete network with all topics included. The size of each circle is indicative of the number of citations from articles within our database for that particular publication

The shape of the network depicted in Fig. 7 suggests three predominant assemblages of topics. One incorporates Cognitive issues, Perception and Navigation, and Simulation (Fig. 7a right). A second demonstrates an assemblage of publications in the area of Neural impact (Fig. 7a left). As shown in Fig. 7b, Interventions, Neurological Conditions, and Gait and Balance are deeply interspersed with Neural Impact; Gaming tends to form a separate assemblage even though its publications permeate the other topics (as would be expected from a term that is widely used in many areas of medicine and science). The relatively few overlapping nodes or linked edges between Psychological issues and the other topics (Fig. 7c) suggests that publications from the topics of Cognitive Issues, Simulation, and Perception and Navigation are more fully linked with each other than those on Psychological issues. Tele-rehabilitation, appearing in Fig. 7d, forms a narrow bridge between those publications focused on either physical (the first assemblage) or psychological (the second assemblage) rehabilitation. The organization of the network implies that publications in the fields of physical and psychological rehabilitation remain separate and demonstrate a low rate of mutual citations even though they employ a similar technology for intervention.

## Discussion

VR-based rehabilitation has traditionally been viewed as an interdisciplinary arena requiring the interaction of computer scientists and engineers with behavioral and clinical scientists (ref). As the technology and software have progressed, research in VR-based rehabilitation has undergone a rapid transformation from having a primary focus on the development of the technology to evaluating how these technologies can be applied toward rehabilitation research and clinical applications. Such changes have been driven mainly by commercial gaming and simulation technology with an increasing number of applications developed at the behest of clinical practitioners and supported by numerous “startups” (https://www.eu-startups.com/2019/02/10-european-vr-startups-to-look-out-for-in-2019-and-beyond/).

The fundamental question in this study was whether research in VR-based rehabilitation may be viewed as a new scientific field within the domain of rehabilitation. Typically, the emergence of a new scientific field has been shown to be driven by the discovery of something new – a central problem and goals and expectations about the solution to the problem – with theories that address these goals [36]. The goal for most VR-based rehabilitation is the achievement of rehabilitation outcomes with the expectation that the use of the particular properties of virtual reality technology will promote clinical gains. But a scientific field can also be described by “a collection of individuals with a common interest in some aspect of science who interact on a regular basis.” ([37], pg. 2). The responsibility of a field is to maintain up-to-date information and provide normative standards for scientific work [37]. Thus the definition of a scientific field depends not only on scientific content but also the extent of scientific interactions.

In order to identify whether the application of VR to rehabilitation exists as a scientific field, we have pursued four questions that we predicted to be answered in the positive through publications analyzed by topic modeling and network analysis. Traditionally, when researchers have tried to understand general trends, topics, or themes in a scientific field, they used systematic reviews and meta-analyses. The selection of publications included in such approaches is often limited by a lack of consistent domain terminology that biases the literature search. There is difficulty in dealing with a large corpus of publications and the scientific discipline and agenda of an individual investigator often directs the focus of analysis [38].

A benefit of text mining is the building of a taxonomy that functionally describes the extensive focus of publications emerging in a multidisciplinary field. This is important to VR-based rehabilitation due to the lack of formal or systematic terminological conventions in this multidisciplinary field [39, 40]. Take, for example, the term “balance” used extensively in both psychology and neurophysiology. In the former domain, balance may refer to a quality of life; whereas, in the latter domain, balance indicates the ability to maintain body position in space. The absence of a domain specific taxonomy limits the ability to study and disseminate intervention techniques [41], and is, in turn, a barrier to identifying outcomes and effectiveness research [42, 43]. Vague taxonomies have been repeatedly cited as a weakness in the rehabilitation literature in general [44], and, potentially, thwart the development of virtual reality-based rehabilitation as a content area within the field of rehabilitation. Topic modeling, a method that analyzes terms within the context of their usage, enables us to look at the terminology in a more nuanced way. For example, the term “balance” will be placed in different topics based on the way it is used within the context of different publications and domains.

Our first question was whether a consistent terminology has emerged in the field of VR-based rehabilitation that could be used to identify the field’s research corpus. The overlap of terms across different topics suggests that virtual rehabilitation has a shared terminology across the engaged scientific and clinical disciplines although specific terms are differentially weighted. Except for the subject category of Psychological issues, the terms “virtual” and “reality” did not emerge as essential terms across all of the topic terms identified in this study (see Appendix). For example, over the years, a range of additional terms including “virtual gaming”, “virtual environments”, “simulated environments” and many others have also been used. Moreover, terminology specific to human factors in virtual reality, for example “presence” and “immersion”, did not appear across all topics.

This finding suggests that a consistent terminology has not been established which may have hindered the formation of an identifiable scientific field. Indeed, the terms that are shared across topics are not specific to a new field of VR-based rehabilitation but can be found in publications from many domains. For example, the term “technology” and terms that we would expect to appear when focusing on healthcare and human behavior (e.g., assessment, patients and performance) are evenly distributed across all topics.

Our second question was whether the collection of terms found in VR-based rehabilitation publications can be used to identify the foremost research corpus in the field. The diverse academic origins of the investigators involved with this technology promotes the use of differing terminology so that even a discussion of similar ideas may be obscured by the use of different terms. The finding, however, that there is no ubiquitous terminology associated with this field alone supports the conclusion that there is no emergent discipline of VR-based rehabilitation. Rather, investigators from many domains share a mutual interest in using these technologies to probe, evaluate and promote innovative rehabilitation applications. Thus, the answer to our second question leads us to conclude that the field of VR-based rehabilitation represents a network of scientific communities with a shared interest in techniques and methods for research and intervention rather than a directed and focused research field.

Scientific fields thrive on collaboration between researchers in technology, cognitive sciences, and health sciences, and the usage of common terminology is important if the collaborative efforts are to eventually form unique academic disciplines [45]. Our results suggest that our third question about whether VR-based rehabilitation has emerged as a well-defined and highly visible multidisciplinary field must be answered in the negative. While multiple disciplines have joined together with a common mandate of applying novel technologies to rehabilitation, the community exists through *interlinked* networks rather than *a single, cohesive field of study*.

These interlinked sub-communities do not imply weakness in the quality or quantity of the science generated by its researchers. Rather, VR-based rehabilitation appears to thrive as a network of traditional disciplines utilizing a common technology rather than as a new multidisciplinary field. Sharing of methods, results and conclusions across investigatory domains occurs when there is an identified need or scientific rationale, but there is no trend, nor indeed, necessity to identify an independent discipline. For example, even though a group of investigators may identify with the subject category of Gait and Balance, the particular research study might be identified by the mechanisms underlying the behavior (e.g., neural vs. biomechanical), the research population being studied (e.g., stroke), the technology employed (e.g., movement analysis), or even the long-term goal of their research (e.g., treatment outcomes). Thus, publications could frequently appear in categories in addition to the primary category of Gait and Balance (e.g., Neural Impact and Neurological Conditions).

### Future directions of this field

Our last question focused on delineating distinguishable areas of research and clinical application that could guide us toward future directions of VR-based rehabilitation. One of the central results from the analysis presented in this paper is that the distribution of publications across topics has varied over time. Subject categories emerging from the WoS search in Period 1 were directed principally toward expanding the technology outside the laboratory. These topics decline greatly in Periods 2 and 3 and physical rehabilitation and intervention become predominant, which might well reflect increased accessibility of the technology for the clinical domain. But if we were to rely on a conventional WoS search, we may have concluded that the categories of rehabilitation, neuroscience, and engineering remain robust across time. By engaging in topic modeling, we have been able to further refine the precise areas of study within those broad categories, and to explore increased productivity and focus over time.

The citation network presented in Fig. 7 illustrates the interactions between the key topics that have emerged in the field of VR-based rehabilitation. For example, many topics interact with the topic “Intervention”. Although publications from the fields of physical and psychological rehabilitation are not directly linked, telerehabilitation bridges cognitive issues and perception/navigation with topics in the physical rehabilitation domain. Across the examined period of time there is evidence that application of this technology has become more widespread throughout the rehabilitation domains, and there are an increased number of investigators and clinicians employing this technology. The paucity of overlapping citations and dissemination of results across the physical and psychological rehabilitation domains, however, implies that contributors continue to communicate and identify more with their original scientific or clinical bases than with the emerging discipline referred to as VR-based rehabilitation. We suggest that awareness of this discrepancy may encourage researchers to search for information in, for example, conference venues that they would not typically access.

### Limitations of the study

This study was exploratory in nature and has several limitations. First, while a leading database served as the data source, it likely does not cover the entire spectrum of publications in the field. This limitation is also an advantage since publications in the WoS database are generally considered to be of high quality and high importance to the scientific community [38]. Another limitation may be that this is a descriptive study which does not support inference at least in its formal connotation of testing hypotheses; for example, we cannot determine why specific terminology has not developed over the years or why communities are interlinked rather than interdisciplinary.

A third limitation relates to the iterative approach of the modeling process between computer-based searching and investigator-driven decision making; we were particularly sensitive to the possibility of a limitation related to bias when selecting the Subject Categories and identifying the topic names. Although the labeling of the topic titles were assigned by the domain experts and, as such, are somewhat subjective, the publications within each topic were automatically extracted and filtered by topic modeling methods and the labels themselves were cross-checked between the significant terms and publications of each topic.

## Conclusions

In order to retain technological currency and rehabilitative impact, research and intervention with VR-based technology requires that the technology and clinical based disciplines communicate their needs and their limitations. However, in order for this communication to succeed, there needs to be a balance between language consistency and academic diversity. The scarcity of interprofessional or interdisciplinary teams participating in clinical trials and technology development underlies the poor reliability and generalizability of results reported by numerous systematic reviews (e.g., [46–49]). Stronger language uniformity, for example in the selection of keywords, will build a more coherent and tightly-knit, albeit interlinked community. Guiding principles for applications of this technology to specific psychological and motor factors in rehabilitation would likely increase the validity and generalizability of outcomes. A team-based, interprofessional approach to rehabilitation and research would significantly strengthen the impact of this technology from the science to its application [50, 51].

Perhaps the most important message emerging from this analysis is the impact these findings might have on the future of VR-based rehabilitation. If our hypothesis that the emergence of VR-based rehabilitation as a distinctive scientific domain requires its proponents to disseminate both research insights and future directions across multiple disciplines is correct, then it is currently unlikely that this field will progress to the level of a scientific discipline. As the technology increases its commercial availability, there is a likelihood that VR technology will be adopted without addressing its strengths and limitations. Often, appreciation of how to optimize human factors or justify selected technological features may be lacking.

In summary, topic modeling was instrumental in cleaning the initial, comprehensive search data and, in the final analysis, it highlighted the research emphases of VR in rehabilitation. Using this approach enabled us to research a large body of documents without needing to reduce the number of items for subjective processing. Being agnostic to publication venue and author status supported the discovery of topics that are central to the field, whether intended or not. This type of analysis brings scientific terminology to the forefront, a topic seldom discussed. In an era where search technology is an obvious tool for research, researchers seeking to build schools of thought are advised to acknowledge the centrality of nomenclature. Professional knowledge and experience led us to some reasonable suggestions, however, they cannot be statistically validated. We are also unable to predict how the data will evolve into the future. The key contribution of this study is to propose recommendations about how to continue to develop a strong research community to ensure valid and robust outcomes from the use of VR technology for rehabilitation research and clinical practice.

## Data Availability

The datasets used and/or analyzed during the current study are available from the corresponding author on reasonable request.

## Appendix

**Table 4:** Probability distribution of terms over topics

Neurological conditions		Cognitive rehabilitation		Simulation		Gait & Balance		Gaming	
Term	Probability	Term	Probability	Term	Probability	Term	Probability	Term	Probability
studies	0.0347	Cognitive	0.0396	simulation	0.0425	balance	0.0633	games	0.0646
effects	0.0313	Task	0.0332	measures	0.0329	control	0.0400	exercise	0.0416
intervention	0.0296	Performance	0.0220	assessment	0.0303	gait	0.0379	children	0.0359
Trial	0.0296	brain	0.0215	performance	0.0293	walking	0.0319	activity	0.0261
rehabilitation	0.0278	learning	0.0214	injury	0.0287	postural	0.0204	physical	0.0241
included	0.0231	skills	0.0190	driving	0.0203	effects	0.0189	wii	0.0232
evidence	0.0196	impairment	0.0182	validity	0.0148	training	0.0150	video	0.0196
quality	0.0166	assessment	0.0167	reliability	0.0146	falls	0.0139	cerebral	0.0167
therapy	0.0162	memory	0.0163	time	0.0143	speed	0.0135	palsy	0.0150
outcome	0.0149	function	0.0146	tests	0.0134	vestibular	0.0120	play	0.0144
systematic	0.0117	ability	0.0131	model	0.0131	dynamic	0.0118	motivation	0.0138
clinical	0.0113	attention	0.0131	evaluation	0.0112	treadmill	0.0117	fit	0.0131
based	0.0099	deficits	0.0117	traumatic	0.0106	adults	0.0116	age	0.0112
control	0.0095	people	0.0104	brain	0.0103	disease	0.0110	level	0.0105
limited	0.0092	executive	0.0101	rate	0.0094	stability	0.0108	exergames	0.0103
data	0.0089	disability	0.0093	correlation	0.0091	Parkinson	0.0092	feasibility	0.0100
improve	0.0087	spatial	0.0092	setting	0.0089	time	0.0089	Kinect	0.0091
treatment	0.0087	navigation	0.0089	TBI	0.0080	increased	0.0086	participation	0.0085
search	0.0078	social	0.0086	objective	0.0076	improve	0.0084	Nintendo	0.0084
physical	0.0078	injury	0.0083	spinal	0.0075	muscle	0.0084	adults	0.0081
Interventions		Neural impact		Perception/Navigation		Psychological issues		Tele-Rehabilitation	
term	Probability	term	Probability	term	Probability	term	Probability	term	Probability
training	0.0594	motor	0.0725	visual	0.0387	virtual	0.2631	rehabilitation	0.0687
patients	0.0535	stroke	0.0494	feedback	0.0263	reality	0.1102	system	0.0608
improve	0.0417	upper	0.0418	motion	0.0210	environment	0.0488	technology	0.0309
stroke	0.0363	movement	0.0388	task	0.0184	real	0.0209	design	0.0224
rehabilitation	0.0298	limb	0.0324	neglect	0.0150	pain	0.0200	based	0.0215
function	0.0262	hand	0.0271	movement	0.0142	patients	0.0151	developed	0.0201
week	0.0234	function	0.0265	healthy	0.0141	disorders	0.0149	computer	0.0178
control	0.200	arm	0.0257	kinematics	0.0131	immersive	0.0115	device	0.0145
therapy	0.0197	rehabilitation	0.0237	reaching	0.0124	body	0.0101	interactive	0.0142
program	0.0190	extremity	0.0218	force	0.0115	training	0.0096	user	0.0141
effects	0.0180	therapy	0.0203	adaptation	0.0114	experience	0.0094	applications	0.0124
intervention	0.0178	activity	0.0196	head	0.0114	world	0.0089	patients	0.0115
sessions	0.0174	recovery	0.0191	target	0.0108	subject	0.0082	therapists	0.0111
based	0.0169	stimulation	0.0176	conditions	0.0096	objective	0.0074	clinical	0.0102
treatment	0.0159	robot	0.0169	patients	0.0095	enhance	0.0074	assisted	0.0094
significant	0.0145	observed	0.0098	display	0.0082	behavior	0.0065	interface	0.0091
outcome	0.0141	imagery	0.0090	perception	0.0081	system	0.0063	provide	0.0090
trial	0.0135	mirror	0.0082	performance	0.0075	potential	0.0059	evaluation	0.0086
reality	0.0128	induced	0.0079	environment	0.0075	purpose	0.0058	proposed	0.0080
post	0.0124	control	0.0077	degrees	0.0072	exposure	0.0056	telerehabilitation	0.0080
